# Parasitic manipulation and neuroinflammation: Evidence from the system *Microphallus papillorobustus *(Trematoda) - *Gammarus *(Crustacea)

**DOI:** 10.1186/1756-3305-3-38

**Published:** 2010-04-15

**Authors:** Simone Helluy, Frederic Thomas

**Affiliations:** 1 Department of Biological Sciences, Wellesley College, Wellesley, MA 02481, USA; 2 Institut de recherche en biologie végétale, Département de sciences biologiques, Université de Montréal, 4101, rue Sherbrooke est, Montréal, Québec, H1X 2B2, Canada; 3 GEMI/UMR CNRS-IRD 2724, 911, avenue Agropolis, BP 64501, 34394 Montpellier Cedex 5, France

## Abstract

**Background:**

Neuropathological consequences of neuroinflammatory processes have been implicated in a wide range of diseases affecting the central nervous system (CNS). Glial cells, the resident immune cells of the CNS, respond to tissue injury by releasing proinflammatory cytokines and free radicals such as nitric oxide. We explored the possibility that neuroimmune responses are involved in parasitic manipulation of host behavior in a trematode-crustacean association. The cerebral larva of the flatworm *Microphallus papillorobustus *alters responses to environmental stimuli - and thus reflex pathways - in the crustacean *Gammarus insensibilis*, in a way that enhances predation of the crustacean by birds, definitive hosts of the parasite.

**Results:**

Immunocytochemical experiments followed by confocal microscopy were performed to study the distribution of glutamine synthetase, a glial cell marker, and nitric oxide synthase in the brain of gammarids. Astrocyte-like glia and their processes were abundant at the surface of the parasites while levels of nitric oxide synthase were elevated at the host-parasite interface in the brain of gammarids harboring mature cerebral larvae and demonstrating altered behavior.

**Conclusion:**

Taken together these results lend support to the neuroinflammation hypothesis whereby a chronic CNS specific immune response induced by the parasite plays a role in the disruption of neuromodulation, neuronal integrity, and behavior in infected hosts.

## Background

Some parasites alter the behavior of their intermediate host in a way that favors the predation of the intermediate host by the definitive host of the parasite, thereby enhancing transmission [see reviews in [1-4]]. Such cases are referred to succinctly as parasitic manipulation [5,6]. Relatively few studies have investigated the proximate mechanisms through which trophically transmitted parasites alter their host behavior. Here, we suggest that specific defense responses of the central nervous system are implicated in the aberrant behavior induced by a cerebral trematode in a crustacean. The larva (metacercaria) of the trematode *Microphallus papillorobustus *(Rankin 1940) encysts in the brain of the crustacean *Gammarus insensibilis *(Stock 1966) and changes the responses of the gammarid to various environmental stimuli, in particular photic, geotactic, and mechanical stimuli [7-10]. The resulting aberrant escape behavior leads to increased predation by birds, the definitive hosts of the parasite [11]. It is important to stress that the parasite does not just induce sluggishness or a general pathological state in the gammarid host. It impinges on the nervous system and alters reflex arcs. Only very specific behaviors are modified. In addition, the larvae are not inducing behavioral alterations from the start of the infection. It is only after a few weeks when the metacercariae are mature and infective to the definitive hosts that the behavioral responses are changed [8] - a common delay in systems involving parasitic manipulation [12]. Therefore, the trematode is modulating the behavior of its host with precise timing and in very subtle ways.

Some acanthocephalans and cestodes [e.g. [13]] present in the hemocoel rather than in the brain of gammarids also modify the behavior of their intermediate hosts. The acanthocephalan *Polymorphus paradoxus *changes the photic and escape behaviors of *Gammarus lacustris *in much the same way as *M. papillorobustus *in *G. insensibilis *[14,15]. In both cases the definitive hosts are birds. Other invertebrates, but also vertebrates, are the subjects of parasite-induced altered responses to environmental stimuli. For example, the protozoan *Toxoplasma gondii *induces in rodents a specific attraction to the odor of cat urine [[16,17]; see also [18] for example on rabies virus in mammals].

While the ecological and evolutionary implications of parasitic manipulation have drawn considerable attention, the neural basis of the altered behavior remains poorly understood [see reviews in [6,19-21]]. It is however established that the serotonergic system is altered in manipulated gammarids harboring acanthocephalans [22-24] as well as trematodes [25,26]. Serotonin modulation has been demonstrated in parasitized vertebrates as well. The concentration of serotonin and other neurotransmitters is selectively altered in parts of the brain of fish infected by cestodes [27] and trematodes [28], and of rodents infected by the nematode *Trichinella spp *[29,30].

This study focuses on the biochemical events upstream of the neurotransmitter dysfunction. Accumulating evidence on the neuropathological consequences of neuroinflammation in vertebrates, coupled with a growing awareness of the common properties of the innate immune response in the vertebrate and invertebrate central nervous systems (CNS), lead to the following hypothesis: The cerebral larva of *M. papillorobustus *causes chronic inflammation in the brain of gammarids, and the pathology associated with this immune response is involved in the neuromodulation and in the altered responses to environmental stimuli manifested by infected gammarids.

The CNS lacks the adaptive arm of the immune system and relies on an innate system involving resident glial cells. In vertebrates, activated glial cells respond to tissue injury by releasing a complex array of inflammatory factors that act on, and engender responses in target cells. Activated glia are known to mediate chronic neuroinflammatory responses that are associated with neurodegeneration and neurological disorders through the release of proinflammatory cytokines, nerve growth factors, and free radicals such as nitric oxide [e.g. [21,31-35]]. Neuropathological consequences of neuroinflammatory responses have been implicated in a wide range of diseases of the nervous system from Parkinson's disease [e.g. [36]], to HIV, multiple sclerosis, Alzheimer's disease [e.g. [37,38]], and rabies [e.g. [39]], as well as in parasitic diseases involving the cerebral larvae of flatworms. For example, the larva of *Taenia solium *(Cestoda) causes neurocystercosis, a common parasitic disease of the human central nervous system worldwide. Much of the pathology of neurocystercosis (epilepsy, chronic headaches) is attributed to the host immune response to the larva in the brain [e.g. [40]]. Immune cascades similar to those observed in vertebrates have been described in invertebrates [41-45]. Thus, the system *M. papillorobustus/G. insensibilis *stands to help our understanding of the debilitating conditions mentioned above, as it represents a simple invertebrate model of chronic cerebral parasitic disease.

The possibility that parasites influence neuromodulation and thus host behavior through the activation of their host's immune response has been invoked in a number of studies over the past decade [reviews in [6,19-21,46-49]]. Behavioral changes in a fish have been connected to the systemic immune response induced by an hemocoelian cestode parasite (*Schistocephalus solidus*) [50]. Ultimately, at least in instances of altered reflex pathways, neuronal disruption has to be involved in host behavioral alteration (e.g. changes in neurotransmitter release and receptor distribution, neurodegeneration of specific pathways, etc.). To our knowledge, the effects of parasites on neuroimmune function in the brain of manipulated hosts have not been tested and are the focus of this research. Immunocytochemical experiments followed by confocal microscopy were performed to find evidence of two main components of the neuroinflammatory response: glial cells and nitric oxide associated with the larva of *M. papillorobustus *in infected brains of *G. insensibilis*.

## Methods

Infected and uninfected brains were incubated in solutions of commercially available antibodies raised against glutamine synthetase (GS), a glial cell marker, and against nitric oxide synthase (NOS), an enzyme that catalyzes the oxidation of L-arginine to produce L-citrulline and nitric oxide (NO). An understanding of the pathology induced by the metacercaria of *M. papillorobustus *can only be achieved through a detailed knowledge of the various regions of the gammarid brain. Therefore the fluorescent label propidium iodide was used to visualize and study the nuclei of cerebral cell populations.

### Gammarid collection and characterization of populations

The life cycle of *Microphalus papillorobustus *necessitates two intermediate hosts (a mollusk of the genus *Hydrobia *and a crustacean of the genus *Gammarus*), and a definitive host, a bird, which harbors the adult intestinal parasite. Two free stages, egg and cercaria infect the mollusk and gammarid respectively [51].

*Gammarus insensibilis *were collected in the brackish waters of the south of France (Etang de Thau, 43°25'N, 3°35'E) in June 2007, September 2007, and June 2008. Gammarids with altered behavior were gathered at the surface of the water near the shore [see for instance [52]]. Normal gammarids were harvested in stacks of algae at the bottom of the lake (about 1.5 m deep). The June 2007 and 2008 samples were sent live to the USA, and dissected and processed for immunocytochemistry at Wellesley College, Massachusetts. The September sample, the largest one, was dissected at the Institut de Recherche pour le Development, Montpellier, and transported in 0.1 M phosphate buffer to the Wellesley laboratory for immunocytochemical processing.

**Figure 1:**
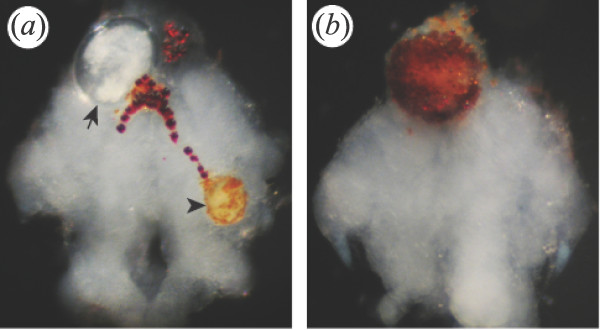
**Larvae of *Microphallus papillorobustus *in whole mount brains of *G. insensibilis***. **(a) **The arrow indicates a mature metacercaria encysted in the protocerebrum, while the arrowhead points to a young larva partially melanized in the deutocerebrum of a gammarid *with altered behavior (MAD)*. Red lipidic granules are seen at the surface of the brain. **(b) **A dead metacercaria is encapsulated and melanized in the protocerebrum of a *normal *gammarid. Anterior is up in these whole mounts viewed with a stereomicroscope. The brains are approximately 1 mm wide.

A simple behavioral test, knocking on the side of the glass aquarium, was used to ascribe each gammarid to one of two categories: "altered behavior" if the animal swam to the surface toward the overhead light, or "normal behavior" if the animal did not swim to the surface following disturbance. For each brain of the September sample, the following characteristics were noted: behavioral status, presence of metacercariae of *M. papillorobustus *in the brain, presence of metacercariae belonging to various species in the body (*M. papillorobustus*, *Maritrema subdolum*, and other microphallids), and presence of nematodes including *Gammarinema gammari *(Table 1). All the gammarids with abnormal behavior were infected with at least one fully-developed cerebral metacercaria of *M. papillorobustus *but harbored up to 13 cysts (length of cysts: 280 to 330 μm; thickness of cyst wall: 15 to 25 μm). A large proportion of normal gammarids (38%) was also infected with cerebral metacercariae, young ones with thin cyst walls, but also larvae encapsulated and melanized (Fig. 1), and larvae apparently mature. Signs of melanization were observed in 13% of the brains (n = 115) but were not seen in the abdomen.

**Table 1:** Characterization of helminth populations in the September gammarid sample.

Gammarids' behavioral status	Total number of gammarids	Gammarids with metacercariae of *M. papillorobustus *in brain	Gammarids with metacercariae of various species in thorax and abdomen	Gammarids with nematodes
		(%)	(%)	(%)
MAD	55	100	69	62

Normal	60	38	30	70

Immature metacercariae, melanized metacercariae, and metacercariae of *M. papillorobustus *and other trematode species located in the thorax and abdomen do not induce the altered behavior [7,53,54]. Thus, two categories of gammarids are referred to thereafter: "MAD" gammarids (with cerebral Metacercaria and Altered Demeanor), and "normal" gammarids.

### Immunocytochemical protocol

Brains were dissected in cool oxygenated Van Harreveld crustacean saline (per liter, in grams: NaCl, 12; KCl, 0.4; CaCl_2 _2H_2_O, 1.5; MgCl_2 _6H_2_O, 0.25; Na HCO_3_, 0.2; pH, 7.3-7.4). They were fixed in 4% paraformaldehyde in 0.1 M phosphate buffer (PB) overnight. The 4% paraformaldehyde fixative was aliquoted, stored frozen, then thawed just prior to use [55]. Brains were rinsed in 0.1 M PB, immersed in 0.1 M PB with 0.2% Triton X-100 (0.1 M PBTX), incubated at 4°C with the primary antibodies against NOS or GS diluted in PBTX, rinsed in 0.1 M PB and then incubated overnight at 4°C with the secondary antibody diluted in PBTX. Subsequently the brains were rinsed in PBTX then in PB; they were bathed in propidium iodide (Invitrogen, P1304 MP) at 25 mg/ml in 0.1 M PB for 15 minutes. After further rinsing in 0.1 M PB, the brains were mounted in 80% glycerol in 0.1 M PB.

### Glutamine synthetase

A purified mouse anti glutamine synthetase monoclonal antibody (BD Biosciences Pharmingen, 610517) was used as a marker of glial cells. The immunogen for this antibody is the amino acid sequence 1-373 of sheep glutamine synthetase. (Product specific information available at http://www.bdbiosciences.com/ptProduct.jsp?prodId=29070).

Western blots with the anti glutamine synthetase antibody reveal that rat cerebrum lysate displays an immunoreactive band at approximately 45 kDA (product specific information from BD Biosciences); western blots of brain homogenate show a single band at approximately 44 kDa in the crayfish *Procambarus clarkii *[56], and at 42 kDa in the spiny lobster *Panulirus argus *[57].

In the present study, incubation in the glutamine synthetase antibody lasted between 24 and 48 hours at a final dilution of 1:100. A total of 51 brains from three experimental batches were processed. Thirty six brains (18 from MAD gammarids, 18 from normal gammarids) were of sufficient quality to be observed in confocal microscopy (Table 2). An additional 6 brains were tested in the absence of glutamine synthetase antibody but with the appropriate secondary antibody; all staining was abolished.

**Table 2:** Characterization of *Microphallus papillorobustus *populations in gammarid brains used for Glutamine Synthetase (GS) experiments.

Gammarids' behavioral status	Number of brains	Number of infected brains	Number of metacercariae per infected brain Mean (Range)
MAD	18	18	1.8 (1 - 4)

Normal	18	7	1.1 (1 - 2)

### Nitric oxide synthase

To study NOS-like immunoreactivity the gammarid brains were incubated in a polyclonal antibody (anti universal Nitric Oxide Synthase, uNOS) raised in rabbit against synthetic peptide DQKRYHDIFG (uNOS, Affinity BioReagents, PA1-039). This peptide sequence corresponds to residues Q 1113 to G1122 of murine inducible NOS and brain NOS. The uNOS antibody shows broad recognition of NOS isoforms (inducible, neuronal, and endothelial NOS), and extensive species crossreactivity in both vertebrates and invertebrates (product specific information from Affinity Bioreagents available at http://www.bioreagents.com/products/productDetails/productDetails.cfm?catnbr=PA1-039). Preadsorption controls with the immunogen (DQKRYHDIFG) used for the production of the Affinity BioReagent antibody abolishes completely immunolabeling in the stomatogastric ganglion of the crayfish *Cherax quadricarinatus *[58].

In the present study, the uNOS antibody was applied to the brains overnight at a final dilution of 1:200. A total of 67 brains from four experimental batches were processed. Thirty nine preparations (22 from MAD gammarids, 17 from normal gammarids) were of sufficient quality to be observed in confocal microscopy (Table 3). An additional 8 brains were processed in the absence of uNOS antibody but with the appropriate secondary antibody. All specific staining was abolished in these "no primary" controls.

**Table 3:** Characterization of *Microphallus papillorobustus *populations in gammarid brains used for Nitric Oxide Synthase (NOS) experiments, and NOS-IR levels in brains infected with one metacercaria (mc).

Gammarids' behavioral status	Number of brains	Number of infected brains	Number of mcs per infected brain	Number of brains with one mc*	Percent difference in the number of NOS-IR particles**
			Mean (Range)		Mean (Range)
MAD	22	22	2.2 (1 - 7)	8	32.6 (-7 to 83)

Normal	17	5	1.6 (1 - 3)	3	3.4 (-9 to 17)

*one single mature metacercaria in MAD gammarids; one developing or fully-developed metacercaria in normal gammarids

** [(n_i _- n_u_)/n_u_] × 100; n_i _represents the number of NOS-IR particles in the infected region of the brain, and n_u _the number of particles in the contralateral uninfected region in brains with one metacercaria.

NOS-like immunoreactivity (NOS-IR) was found as a punctate signal in the gammarid brain. To quantify this phenomenon, the number of NOS-IR particles was analyzed with the application Image J of the National Institute of Health http://rsbweb.nih.gov/ij/. The number of IR particles was computed in all the infected brains presenting a single mature metacercaria in MAD gammarids (n = 8, 5 with metacercaria in the protocerebrum, 3 in the deutocerebrum), and in brains of normal gammarids with one metacercaria at various stages of development (n = 3, all in the protocerebrum) (Table 3). A paired t-test compared the number of IR particles in the infected side of the brain and in the contralateral region (protocerebrum or deutocerebrum depending on the location of the metacercaria).

### Secondary antibodies

Two secondary antibodies were used following incubation with the mouse anti glutamine synthetase antibody: CY2-conjugated AffinityPure donkey anti-mouse IgG (H+L) (Jackson Immunoresearch, 715-225-150), overnight at a final dilution of 1:100; and Alexa 488 goat anti-mouse IgG (H+L) (Invitrogen, A11029) overnight at 1:50. Alexa 488 donkey anti-rabbit IgG (H+L) (Invitrogen, A21206) was used overnight at a dilution of 1:50 after incubation of the brains in the primary antibody rabbit anti-uNOS.

**Figure 2:**
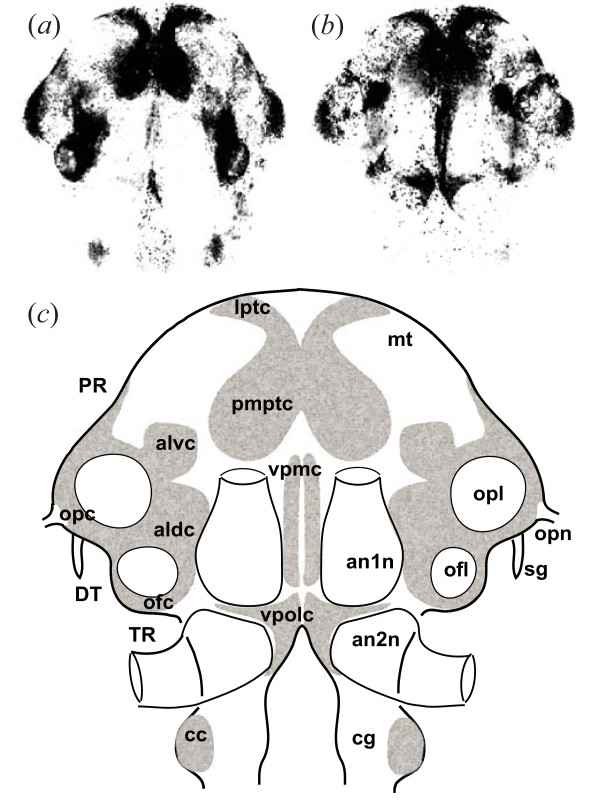
**Brain morphology in *G. insensibilis***. **(a) and (b)** Single horizontal confocal sections in the dorsal and ventral regions of the brain respectively. Cell nuclei stained with propidium iodide appear black (high density of nuclei) or grey (low density). The compound eyes located on either side of the protocerebrum have been removed. **(c)** Schematic outline of prominent cell clusters, and location of neuropil regions. Terminology [adapted from 86] and abbreviations; aldc, anterior lateral dorsal cell cluster; alvc, anterior lateral ventral cell cluster; an1n, antennal 1 neuropil; an2n, antennal 2 neuropil; cc, circumesophageal cell cluster; cg, circumesophageal ganglion; DT, deutocerebrum; lptc: lateral protocerebral cell cluster; mt: medulla terminalis; ofc, olfactory cell cluster; ofl, olfactory lobe; opc, optic cell cluster; opl, optic lobe; opn, optic nerve; pmptc, paramedial protocerebral cell cluster; PR, protocerebrum; sg, sinus gland; TR, tritocerebrum; vpmc, ventral paramedial cell cluster; vpolc, ventral posterior lateral cell cluster. The width of the brain is approximately 1 mm.

Brain whole mounts were viewed with a Leica TCS-NT scanning confocal microscope. The fluorophores Alexa Fluor 488 and CY2 were visualized with an argon gas laser (emission line 488 nm) and propidium iodide was observed with a krypton gas laser (emission line 561 nm) allowing the study of the various cell clusters (Fig. 2).

## Results

### Putative glutamine synthetase immunoreactivity

**Figure 3:**
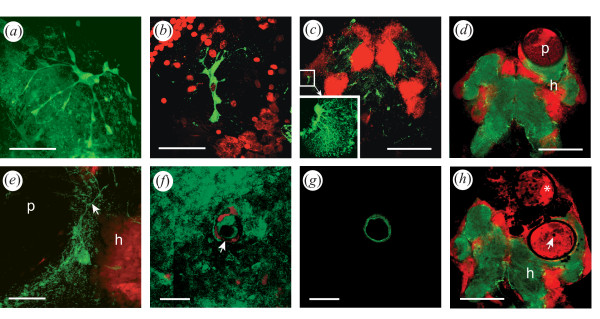
**Putative glutamine synthetase immunoreactivity (GS-IR, green) in the brain of *G. insensibilis***. Cell nuclei are counterstained with propidium iodide (red label). **(a) to (c)** Various glial cell morphologies. Note the end feet and the flocculent profiles of the astrocyte-like cell shown in (a). **(**d** and e)** GS-IR in brains of MAD gammarids. Glial cell bodies are present at the surface of the metacercariae. Fine processes (arrow) are apposed to the cystic wall in this stack of confocal sections through a metacercaria (e). **(f) and (g)** Confocal sections at different levels of an invagination of the cyst wall in a metacercaria. The sections are tangential to the cyst; a section at the surface of the metacercaria (f) shows flocculent glial profiles around the opening of the invagination (arrow); a section taken through the cyst wall reveals the GS-IR wall of the invagination (g); **(h)** Brain of a MAD gammarid with one live and one encapsulated metacercaria. The asterisk indicates a larva encapsulated and presumably moribund. The arrow points to the invagination of the cyst wall presented in pictures (f) an (g) in a second metacercaria. In (c), (f), (g), (h), single confocal sections; in (a), (b), (c insert), (d), (e), stacks of confocal sections. For clarity the propidium iodide counterstain has been omitted in (a) and in the insert of (c). Anterior is up; h, host; p, parasite. Scale bars: (c), (d), (h) 300 µm; (a), (b), (e), 50 µm; (f), (g), 20 µm.

Putative glutamine synthetase immunoreactive (GS-IR) cell bodies were distributed throughout the brain at the level of the neuropils in both MAD (with metacercaria and altered demeanor) and normal gammarids. GS-IR cells showed a variety of morphology: "astrocyte"-like, x- shaped, or globular (Fig. 3). Large astrocyte-like cells spanning more than 100 μm extended fine processes terminating in end feet and flocculent material (Fig. 3a). Other GS-IR cells were more compact (Fig. 3b). The most noticeable glial cell bodies formed a sheath at the surface of the neuropils and extended thin processes within the tangle of neurites, thus staining the entire neuropil. The antennal neuropils were generally the most intensely immunoreactive whereas the olfactory lobes were less densely labeled. A pair of prominent identifiable cells was present in every individual laterally on either side of the brain, projecting fine extensions toward the center of the optic lobes (Fig. 3c).

In MAD gammarids, GS-like immunoreactivity was found at the surface of the ovoid cysts (Fig. 3d). The processes of astrocyte-like GS-IR cells were lining the cyst wall of the part of the metacercariae in contact with brain tissues. Filiform (Fig. 3e), or flocculent (Fig. 3f) GS-IR profiles, were found apposed to the surface of the parasite. In some cases, a meshwork of thin processes invaded the intercellular space between the neuronal somata adjacent to the cyst wall. Confocal sections tangential to the cyst wall showed that GS-IR was also present in invaginations extending into some of the metacercariae (Fig. 3f and 3g). The tissues surrounding encapsulated larvae appeared devoid of GS-IR cells or processes (Fig. 3h). In this figure, one metacercaria was partially encapsulated but alive and still located amidst some living tissues as shown by the presence of the nucleic acid label propidium iodide within and around the larva. Eventually, only black areas mark the location of dead encapsulated larvae.

### Putative nitric oxide synthase immunoreactivity

**Figure 4:**
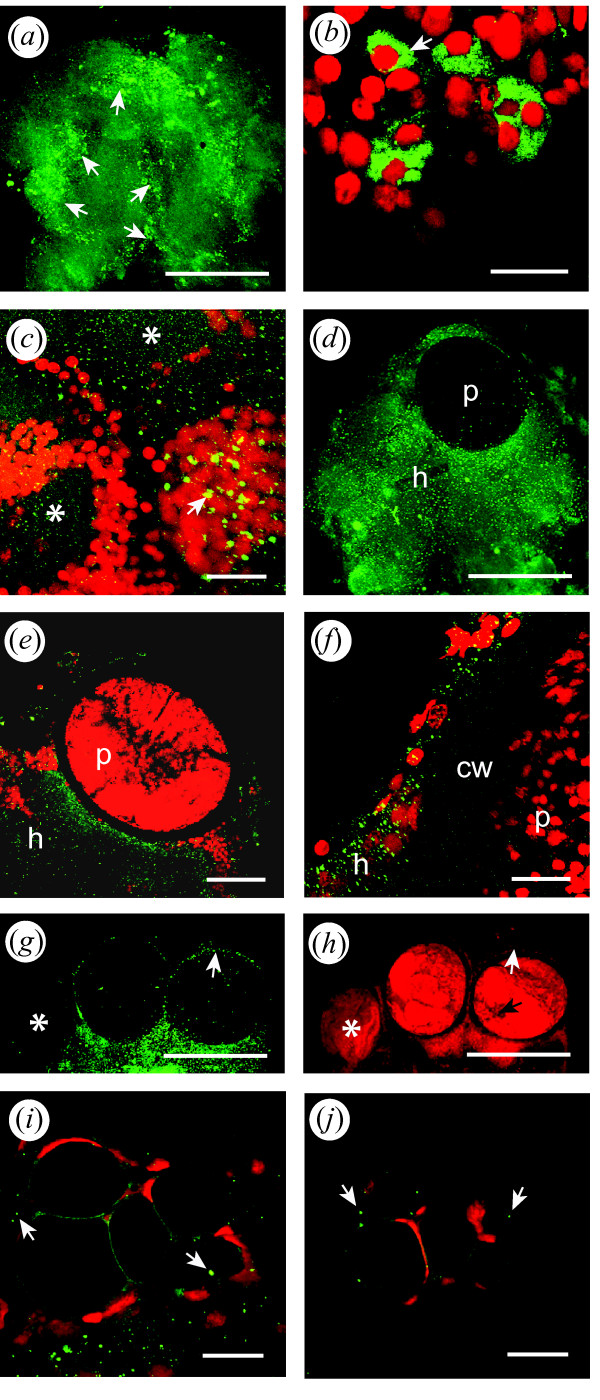
**Putative nitric oxide synthase immunoreactivity (NOS-IR, green) in the brain of *G. insensibilis***. **(a) **Entire brain showing the bilateral NOS-IR distribution. The signal is concentrated at the level of the cell clusters pmptc, alvc, ofc, vpolc, and vpmc (arrows, see Fig. 2 for abbreviations). **(b) and (c)** NOS IR in somata and neuropils. NOS-IR is localized in the somata of neuronal cell clusters [arrows in (b) and (c)]. NOS-IR is also found in neuropil regions as a punctuate signal [asterisks in (c)]. **(d to f)** Metacercariae in the protocerebrum of MAD gammarids surrounded by intense punctate NOS-IR. **(g) and (h)** One juvenile (asterisk) and two mature metacercariae in the protocerebrum of a MAD gammarid. The NOS label is shown in (g) whereas the propidium iodide signal is seen in (h); the white arrows indicate NOS-IR tissues present around a mature metacercaria but not around a young larva (asterisk). The black arrow points to the invagination of the cyst wall displayed in pictures (i) an (j). **(i) and (j)** Confocal sections at different levels of an invagination of the cyst wall in a metacercaria. The sections are tangential to the cyst; the arrows point to NOS-IR particles. In (b), (e), (g), (h), (i), (j), single confocal sections; in (a), (c), (d), (f), stacks of confocal sections. For clarity the propidium iodide counterstain has been omitted in (a) and (d). Anterior is up; cw, cyst wall; h, host; p, parasite. Scale bars: (a), (d), (g), (h), 300 μm; (e), 100 μm; (c), 30 m; (b), (f), (i), (j), 20 μm;.

Putative NOS-immunoreactivity (NOS-IR) was distributed throughout the brain of both MAD and normal gammarids (Fig. 4). NOS-IR was concentrated at the level of specific bilateral cell clusters: paramedial protocerebral cell cluster (pmptc), anterior lateral ventral cell cluster (alvc), anterior lateral dorsal cell cluster (aldc), olfactory cell clusters (ofc), and ventral posterior lateral cell cluster (vpolc), forming a ring in the brain (Fig. 4a; see Fig. 2 for gammarid brain anatomy). The fluorescent label was also in evidence in the ventral paramedial cell clusters (vpmc) along the sagittal axis of the brain. The intensity and the appearance of the NOS label were highly variable. In some brains the label was concentrated at the level of the cell clusters as described above (Fig. 4a); some punctate label was also visible. In other brains the typical distribution at the level of the cell clusters was clear, but the signal was more diffuse and presented a wide-spread punctate appearance. The more concentrated label corresponded to neuronal somas (approximate diameter: 10 to 15 μm) or to small groups of somas in which the cytoplasm was filled (Figs. 4b) or partially filled (Fig. 4c) with NOS immunoreactivity. The punctate label present at the level of the neuropils was formed by particles measuring between 1 and 3 microns.

The distribution of NOS-IR was similar in the brains of MAD and normal gammarids. However, the intensity of the NOS punctate label was increased in the tissues adjacent to mature metacercariae in the brain of MAD gammarids (Fig. 4d to 4j). On average, the number of IR particles was 33 percent higher in the infected region than in the contralateral uninfected region in the 8 brains of MAD gammarids presenting a single mature metacercaria (Table 3). The difference in the mean number of NOS-IR particles between the two regions was significant (paired t-test, df = 7, t = 2.64, p = 0.03). In the few normal gammarids harboring one metacercaria (n = 3), the number of IR particles was only 3.4 percent higher in the infected region than in the contralateral uninfected region (Table 3). The difference between the two regions was not significant (paired t-test, df = 2, t = 0.44, p = 0.70). Moreover, young larvae developing in the brain of MAD gammarids (n = 5) did not appear to be surrounded by increased levels of NOS-IR (Fig. 4g and 4h). The GS positive invaginations within the cyst wall that were observed in some parasites (Fig. 3g) were also NOS positive (Fig. 4i and 4j).

## Discussion

So far, little attention has been given to the potential involvement of neuroinflammatory processes in the altered reflex pathways induced by some parasites in their hosts. In the brain of *M. papillorobustus*-infected gammarids, immunocytochemistry revealed two components of the neuroimmune response: glial cells and nitric oxide. Astrocyte-like glia and their processes lined the cyst wall of the metacercariae, and high levels of nitric oxide synthase were present at the host-parasite interface.

### Nature and significance of the glutamine synthetase signal

The enzyme glutamine synthetase catalyzes the amination of glutamic acid to form the amino acid glutamine. Glutamine synthetase has been used as a reliable marker of glial cells in the vertebrate brain [e.g. [59]] and in crustaceans [56,57,60,61]. However, the neuroimmune role of glia in crustaceans has not been investigated. In *G. insensibilis*, we found large, GS-IR, star-shaped, astrocyte-like cells (Fig. 3a) that exhibit morphological similarities with their counterparts in the Drosophila brain [see review in [62]]. Microglia-like cells, which are GS immunoreactive in mammals [63], were not observed in this study. Again, the small gammarid brain shows similarities with the Drosophila brain. In this insect, a category of glia dedicated to immune functions, such as the mammalian microglia, does not appear to be present. Instead, all glial cells seem competent to perform immune functions [62].

The salt of glutamic acid, glutamate, is an important excitatory neurotransmitter in the vertebrate and crustacean CNS while GABA, downstream in the same synthetic pathway, is the main inhibitory transmitter. In arthropods [reviews in [57,62,64,65]] as in vertebrates, glia plays a role in a variety of functions including the reuptake of presynaptically released neurotransmitters from the synaptic cleft. The enzyme GS is specifically involved in converting glutamate into glutamine, a non neuroactive amino acid. Glutamine is then released by glia and recycled by neurons. In HIV infection, neuronal damage results mainly from glial activation and involves glutamate-mediated neurotoxicity [see review in [66]]. The presence of GS immunoreactive glial cells adjacent to the metacercariae suggests a potential alteration in glutamate metabolism in the brain of infected gammarids, in addition to other functions such as the release of proinflammatory cytokines and free radicals.

### Nature and significance of the nitric oxide synthase signal

NOS was concentrated at the level of the neuronal cell clusters in both normal and MAD gammarids and was found as a more intense signal surrounding the cyst wall. The synthesis of the gaseous signaling molecule NO from L-arginine is catalyzed by the enzyme nitric oxide synthase (NOS). NO acts as a membrane permeant diffuse signaling molecule. The antibody used in the present study, universal NOS, detects the three isoforms of the enzyme: neuronal, endothelial, and inducible. The same universal NOS antibody (Affinity BioReagents, PA1-039), used in the present study revealed NOS immunoreactivity in somata and neuropils in the cardiac ganglion [67], in the stomatogastric ganglion [58] and in the brain [68] of crayfish. In these studies, punctate NOS immunolabeling was observed in the cytoplasm of neuronal cell bodies [58] as well as in neuropil regions [68].

It could be argued that the voluminous metacercariae displace and compress the adjacent tissues in the host brain, and that the higher density of NOS particles surrounding the metacercariae is a consequence of this phenomenon. If this were the case, similar numbers of NOS-IR particles would be found in infected and uninfected regions, with a more widespread signal in the uninfected region unencumbered by the bulky parasite. However, the fact that, on average, the number of NOS particles was higher in the infected region than in the contralateral region of the brain speaks against the "compression" hypothesis and in favor of an actual increase in NOS levels in the presence of the parasite. The variability observed in NOS levels could be accounted for by changing immunogenic properties of the parasite over time [see [50]], and by inter-individual variations in the efficacy of the innate immune system of the host. The variability in the degree of parasite-induced behavioral manipulation has been demonstrated in various host-parasite associations [e.g. [12,24]].

NO has already been implicated in the *M. papillorobustus-G. insensibilis *association. Proteomics studies indicate that arginine kinase - a regulating factor in NO synthesis - is differentially expressed in the brain of *M. papillorobustus*-infected *G. insensibilis *as well as in a gammarid/acanthocephalan system [26]. But neither the proteomics study nor the present study ascertained the origin - glial or neuronal - of the NOS signal at the host-parasite interface. In mollusks, NOS is activated in both microglial cells and neurons in the presence of specific immune challenges [43]. Converging lines of evidence show that bidirectional signaling molecules mediate some interactions between the immune and nervous systems in insects [69].

Whether of glial or of neuronal origin, changes in NO levels could have a variety of effects. NO has been shown to mediate neurotoxicity in some aminergic systems [e.g. [70,71]]. In rodent brains, NO alters the levels of serotonin [72-74], and dopamine [73,74]. NO may also play a general role in the development of discrete neural networks [75]. NO is already known as a systemic immune effector in vertebrates and invertebrates [review in [76]]. The examples cited suggest that increased levels of NO at the host-parasite interface could not only participate in the host defense response but also potentially affect neuromodulation and/or neuronal development in the brain of *M. papillorobustus- *infected *G. insensibilis*.

### Encapsulation of metacercariae and CNS-specific defense responses

In many instances, the immune response in the brain of the gammarid host leads to encapsulation and parasite death. In the sample studied, 13% of cerebral metacercariae showed signs of partial or complete melanization (Fig. 1, Fig. 3h). When the parasite is encapsulated, host behavior is not modified [7,53,54]. The cell population responsible for larval encapsulation in gammarid brains has not been characterized and the components of the cerebral reaction have not been elucidated. What is known is that macroparasites located in the body of arthropods elicit an encapsulation response that involves two elements: the deposition of eumelanin resulting from the prophenol oxydase system activation, and the adhesion of numerous hemocytes around the parasite [reviews in [77-79]]. Encapsulation and melanization always indicate the failure of parasitism. It is conceivable that both the systemic immune response (hemocytes infiltrated through the blood-brain barrier) and endogenous cerebral immune cells (glia) are involved in interactions with the cerebral cysts of *M. papillorobustus *at various stages of the host response to infection. The initial penetration of the perineurium by the cercaria could also have long-term consequences for tissue repair and glial proliferation. Indeed, one month after the surgical lesion of the perineurium, hemocyte-like granule containing cells were found within the brains of cockroaches in the vicinity of the cut surface [80], whereas a stab injury in the brain of *Drosophila *induced prolific glial division [81].

Small developing metacercariae are wrapped in a thin membrane that must expand as the parasite enlarges during metamorphosis from cercaria to metacercaria. Fully-developed metacercariae are contained in a rigid, thick (15 to 25 μm) ovoid cyst wall. In *Microphallus opacus *[82], this final cyst wall is composed of three layers completed in about 30 days. The changing surface of the cyst wall is likely to have different properties with regard to host recognition [50]. Thus, different immune evasion tactics might be deployed by the *M. papillorobustus *larvae over the course of the infection [review in [83]]. The presence of elevated NOS-IR found at the surface of mature metacercariae but not of young larvae suggests that the latter are mostly avoiding detection in the brain of their hosts. Mature metacercariae would trigger a response, but, protected by a sturdy cyst wall, would generally avoid encapsulation and survive the chronic attack of the host immune system. In less resilient metacercariae, the GS-IR (Fig. 3f and 3g) and NOS-IR (Fig. 4i and 4j) positive invaginations penetrating the cyst wall may represent the first signs of weakening of the larvae and herald the demise of the parasites through encapsulation.

Eslin & Prevost [84] investigating a host/parasitoid system suggest the existence of a race between the encapsulation reaction by the hemocytes of the host and the safe embedment of the parasitic eggs within the host tissue. By analogy, the interaction between *M. papillorobustus *and *G. insensibilis *could be envisioned as an arm wrestling contest between cerebral larva and host: If the growing parasite avoids being detected and reaches a mature stage protected by a thick cyst wall, then the host's response becomes a chronic neuroinflammatory condition with neuropathological and behavioral consequences. However, the host innate immune system may at times overcome mature metacercariae (Fig. 3h). The time course of neural events - e.g. switches in enzymatic activity, up and down regulation of neurotransmitters and receptors, synaptic reorganization, axonal sprouting and growth - may unfold over several days. It is therefore reasonable to assume that a delay might exist between inflammatory response and changes in behavior [85].

Further investigations could aim at strengthening the connection between the neuroinflammatory condition and behavioral disruption. For example, a longitudinal study of the NOS-IR signal might reveal the precise timing of neuroinflammatory events in relation to the appearance of the altered behavior. In addition, an ethopharmacological approach, such as injecting MAD gammarids with anti-inflammatory drugs, could help establish the link between the host cerebral immune response and the neural and behavioral pathology. While further evidence is needed to demonstrate the relationship between glia, nitric oxide, and serotonergic dysfunction, the present research constitutes a first empirical step in the exploration of the role of neuroimmune processes in parasitic manipulation of gammarid behavior.

## Competing interests

The authors declare that they have no competing interests.

## Authors' contributions

SH conceived the project, carried out the experiments, and wrote the manuscript. FT conceived the project, led the field work, and critically revised the manuscript. Both authors read and approved the final manuscript.
